# Therapy-related myelodysplastic syndrome rapidly evolving to acute myeloid leukemia following CAR-T cell therapy in a patient with refractory diffuse large B-cell lymphoma: a case report

**DOI:** 10.3389/fonc.2026.1888595

**Published:** 2026-07-14

**Authors:** Longmei Chen, Wanchao Liu

**Affiliations:** Department of Laboratory Medicine, Shanghai Baoshan Hospital of Integrated Traditional Chinese and Western Medicine (Baoshan Hospital, Shanghai University of Traditional Chinese Medicine), Shanghai, China

**Keywords:** CAR-T cell therapy, diffuse large B-cell lymphoma, persistent cytopenia, therapy-related acute myeloid leukemia, therapy-related myelodysplastic syndrome

## Abstract

**Background:**

Persistent cytopenia following chimeric antigen receptor T-cell (CAR-T) therapy is an increasingly recognized complication in patients with relapsed or refractory diffuse large B-cell lymphoma (DLBCL). While most cases stem from transient, immune-mediated mechanisms or depleted bone marrow reserves, secondary therapy-related myeloid neoplasms (t-MN) must be strongly suspected in patients presenting with prolonged, transfusion-refractory cytopenias.

**Case presentation:**

We report the case of a 76-year-old woman diagnosed with refractory DLBCL (non-germinal center B-cell subtype, stage IIIA, International Prognostic Index score of 4). Following multiple lines of chemo-immunotherapy (R-CDOP, R-CDOPE, R-ICE, local radiotherapy, and one cycle of Pola-R-ICE) and subsequent autologous CAR-T cell infusion, the patient developed persistent pancytopenia, characterized by severe, transfusion-refractory thrombocytopenia. A comprehensive bone marrow evaluation performed in February 2026 revealed multilineage dysplasia, 13% blasts, *TP53* deletion/mutation, and a complex karyotype, establishing a diagnosis of therapy-related myelodysplastic syndrome with low blasts (t-MDS-LB). Within two months, the disease rapidly progressed to acute myeloid leukemia (t-AML) with 35% abnormal myeloid blasts. Combination therapy with azacitidine and venetoclax was initiated but discontinued due to profound myelosuppression and severe infection.

**Conclusion:**

This case underscores that persistent post-CAR-T cytopenia can serve as an early manifestation of an underlying t-MN rather than transient hematotoxicity. Prompt and comprehensive bone marrow assessments—incorporating morphology, flow cytometry, cytogenetics, and molecular profiling—are paramount for patients with extensive prior exposure to cytotoxic regimens to avoid catastrophic diagnostic delays.

## 1 Introduction

Diffuse large B-cell lymphoma (DLBCL) is the most common aggressive subtype of non-Hodgkin lymphoma (NHL) (1). Despite substantial improvements achieved with immunochemotherapy, a considerable proportion of patients eventually develop relapsed or refractory (R/R) disease. In recent years, CD19-directed chimeric antigen receptor T-cell (CAR-T) therapy has markedly reshaped the therapeutic landscape for R/R DLBCL, achieving durable complete responses (CR) in approximately 40% of cases (2).

However, as the clinical use of CAR-T therapy expands, long-term toxicities have emerged as a focal point of clinical management (3). Among these, prolonged cytopenia—defined as cytopenia persisting beyond day 30 post-infusion—remains a frequent and challenging complication (4). Although post-CAR-T cytopenia is commonly attributed to lymphodepleting chemotherapy, inflammatory cytokine-mediated suppression, or a depleted hematopoietic reserve, growing evidence indicates that therapy-related myeloid neoplasms (t-MN)—including therapy-related myelodysplastic syndrome (t-MDS) (5) and acute myeloid leukemia (t-AML) (6)—can selectively emerge in a subset of these heavily pretreated individuals. These secondary malignancies are cytogenetically adverse, frequently characterized by *TP53* alterations and complex karyotypes, and carry an extremely dismal prognosis.

Differentiating an evolving t-MN from standard treatment-related hematotoxicity presents a formidable diagnostic dilemma, often leading to fatal clinical delays. While isolated cases of t-MDS or t-AML post-CAR-T therapy have been described in the literature, the documentation of a shift, the sequential evolution from t-MDS to overt t-AML within this specific post-infusion window remains virtually unreported. Here, we report a case of refractory DLBCL in an elderly patient who developed persistent pancytopenia after CAR-T cell therapy, which catastrophically transitioned from t-MDS to overt t-AML within two months, highlighting the critical need for vigilant bone marrow surveillance.

## 2 Case presentation

### 2.1 Initial presentation and diagnostic workup

In April 2024, a 76-year-old woman presented with persistent toothache and facial discomfort. Biopsy and surgical resection of a sinonasal mass were performed. Histopathological examination of the left maxillary sinus lesion revealed diffuse infiltration of atypical lymphoid cells with extensive necrosis. Immunohistochemistry analysis demonstrated positivity for CD20, BCL6, and MUM1, with a high Ki-67 proliferation index (~90%). The tumor cells were negative for CKpan, P63, CD3, CD5, CD10, CD30, CD56, Cyclin D1, and Epstein–Barr virus-encoded RNA (EBER). Based on the Hans algorithm, the immunophenotype (CD10−/BCL6+/MUM1+) was classified as the non-germinal center B-cell (non-GCB) subtype of DLBCL.

The differential diagnosis included T-cell lymphoma, extranodal NK/T-cell lymphoma, mantle cell lymphoma, EBV-associated lymphoma, myeloid sarcoma, and poorly differentiated carcinoma. T-cell lymphoma was excluded by the absence of CD3 expression, extranodal NK/T-cell lymphoma and EBV-associated lymphoma by negative EBER and CD56 staining, mantle cell lymphoma by negative Cyclin D1 expression, and poorly differentiated carcinoma by negative epithelial markers (CKpan and P63). Bone marrow histopathology ruled out acute myeloid leukemia (AML): trilineage hematopoiesis was generally normocellular, with no infiltrative neoplastic lymphoma cells detected in the core biopsy specimen.

Therefore, a diagnosis of diffuse large B-cell lymphoma (DLBCL, non-GCB subtype) was established. Based on comprehensive staging, the patient was classified as stage IIIA with an International Prognostic Index (IPI) score of 4, indicating high-risk disease.

### 2.2 Pre-CAR-T therapeutic timeline

**Figure 1:**
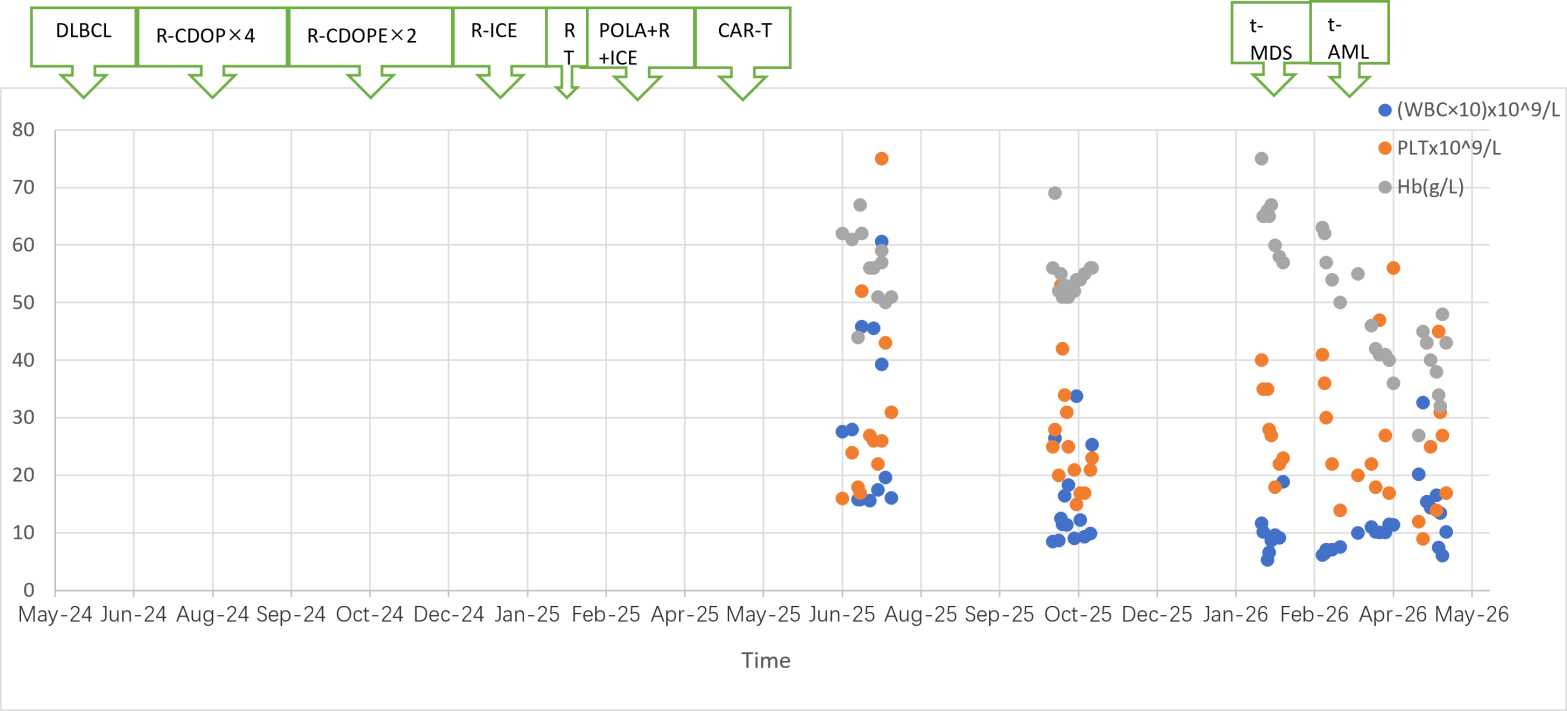
Clinical course and hematologic evolution timeline. DLBCL, diffuse large B-cell lymphoma; R-CDOP, rituximab, cyclophosphamide, doxorubicin, vincristine, and prednisone; R-CDOPE, rituximab, cyclophosphamide, doxorubicin, vincristine, prednisone, and etoposide; R-ICE, Rituximab, ifosfamide, carboplatin, and etoposide; RT, radiation therapy; POLA+R+ICE, polatuzumab vedotin, rituximab, ifosfamide, carboplatin, and etoposide; CAR-T, chimeric antigen receptor T-cell therapy; t-MDS, therapy-related myelodysplastic syndrome; t-AML, therapy-related acute myeloid leukemia; WBC, white blood cell; PLT, platelet; Hb, Hemoglobin.

The patient’s subsequent clinical course was marked by highly chemorefractory disease, necessitating multiple therapeutic switches (**Figure 1**). Given the patient’s poor physical status, the R-CDOP regimen was selected as frontline therapy.

Cycle 1: R-CDOP (Jul 1, 2024): Rituximab 600 mg D6; cyclophosphamide 0.6 g D3 + 0.4 g D4; liposomal doxorubicin 20 mg D4–5; vincristine 4 mg D2; dexamethasone 15 mg D1–5.

Cycles 2–4: R-CDOP (Jul 31, Aug 28, Sep 26, 2024): Uniform day-1 dosing: Rituximab 600 mg D1, cyclophosphamide 1 g D1, liposomal doxorubicin 40 mg D1, vincristine 2 mg D1, dexamethasone sodium phosphate 15 mg D1–5.

Interim PET-CT assessment indicated inadequate disease control. The regimen was shifted to two cycles of R-CDOPE (incorporating etoposide); however, the left facial mass progressively enlarged during treatment.

Cycles 5: R-CDOPE (Oct. 27, 2024): Rituximab 600 mg, Day 0; Etoposide 100 mg, Days 2–4; Cyclophosphamide 1.1 g, Day 2; Liposomal doxorubicin 40 mg, Day 2; Vincristine 2 mg, Day 2; Dexamethasone 15 mg, Days 2–6.

As sequential R-CHOP followed by ICE has been reported to yield high efficacy, this regimen avoids radiotherapy. Importantly, 50% of patients with treatment failure can be salvaged with radiotherapy-based hematopoietic stem cell transplantation. As a result, R-ICE salvage regimen was adopted (7).

Cycles 6 & 7: R-ICE salvage regimen (Dec 04, 2024; Jan 09, 2025): Rituximab 600 mg, Day 0; Etoposide 120 mg, Days 1–3; Ifosfamide 5 g, Day 2; Carboplatin 300 mg, Day 2.

The patient presented with advanced age (>60 years), stage III disease, extranodal involvement (2.5-cm right submandibular lymphadenopathy), Eastern Cooperative Oncology Group (ECOG) performance status (PS) >1, and an International Prognostic Index (IPI) score of 4, consistent with high-risk disease. For patients with an IPI score ≥2, the Pola-R-CHP regimen (polatuzumab vedotin, rituximab, cyclophosphamide, doxorubicin, prednisone) may be considered. The primary lesion within the left upper alveolus measured 6.4 cm × 9.4 cm with a maximum diameter exceeding 7.5 cm, thus involved-site radiotherapy (ISRT) was combined.

Cycle 8: Pola-R-ICE bridging regimen (Apr 02, 2025): Etoposide 60 mg, Days 1–3; Ifosfamide 2 g, Day 2; Carboplatin 150 mg, Day 2; Rituximab 0.6 g, Day 3; Polatuzumab Vedotin 90 mg, Days 3–4.

Radiotherapy details

1. Irradiation anatomical target volume

The clinical target volume (CTV) encompassed the primary hypermetabolic lesion of the left maxillary sinus with contiguous invasive structures (left nasal cavity, ethmoid sinus, orbital floor, facial soft tissue, pterygopalatine fossa, infratemporal fossa, retromaxillary space, upper alveolar region), as well as involved left cervical and bilateral submandibular lymph nodes identified on baseline PET-CT. Organs at risk including left lens, optic nerve, brainstem and spinal cord were delineated for dose sparing.

2. Total prescribed radiation dose

The planned salvage radiotherapy total dose was 40 Gy (to be supplemented per radiotherapy chart).

3. Fractionation regimen

Conventional fractionation scheme: 2 Gray (Gy) per fraction, once daily, 5 days per week.

4. Total fractions planned & delivered

A total of 20 fractions were scheduled; 9 fractions were completed by mid-March 2025. Post-radiation skin adverse event: soybean-sized skin lesion developed suborbitally within the irradiated field, which progressed to a chronic sinus tract requiring regular normal saline irrigation.

Cycle 9: Lymphodepletion Conditioning: Prior to CAR-T cell infusion, the patient received a fludarabine–cyclophosphamide (FC) lymphodepletion regimen consisting of fludarabine 50 mg/day and cyclophosphamide 0.75 g/day for three consecutive days. Autologous anti-CD19 CAR-T cell infusion was performed on May 08, 2025, with a total infused cell suspension volume of 68 mL per bag. The target cell dose was 2×10^6^ anti-CD19 CAR-T cells per kilogram of body weight, corresponding to a total cell count of 1.1×10^8^ cells in one single bag. The infusion was uneventful, without adverse reactions such as fever or rash observed. Granulocyte colony-stimulating factor (Recombinant Human G-CSF, Ruibai) was administered for supportive care during the myelosuppression phase.

### 2.3 Post-CAR-T persistent cytopenia

During the post–CAR-T follow-up period, the patient experienced an episode of suspected urinary tract infection on June 17, 2025, presenting with fever, urinary frequency, and urgency, which resolved after anti-infective therapy. In addition, the patient developed herpes zoster involving the right lower limb on October 3, 2025, without an identifiable trigger, suggestive of persistent post–CAR-T immune dysregulation. No severe cytokine release syndrome or immune effector cell-associated neurotoxicity syndrome was observed during the early post-infusion period.

In addition, the patient experienced severe and prolonged pancytopenia. While her white blood cell count (WBC) demonstrated transient, fluctuating improvements following the administration of granulocyte colony-stimulating factor (G-CSF), her thrombocytopenia was profoundly refractory to supportive care, with platelet counts (PLT) consistently remaining below 20×10^9^/L. She received intermittent supportive care consisting of recombinant human G-CSF and hetrombopag. Due to the unexplained, prolonged duration of hematotoxicity, diagnostic bone marrow evaluations were initiated.

### 2.4 Hematopathologic and cytogenetic evaluation (february 2026)

A comprehensive bone marrow (BM) assessment was performed on February 26, 2026, to investigate the persistent cytopenia:

#### 2.4.1 BM aspirate morphological examination

The smear revealed a markedly hypocellular marrow with a myeloid-to-erythroid (M:E) ratio of 0.6:1. Blasts accounted for 13% of all nucleated cells. The granulocytic lineage showed decreased proliferation with a left shift, accompanied by overt dysplastic features including increased coarse cytoplasmic granules and hypolobated mature neutrophils (pseudo-Pelger-Huët anomaly). The erythroid lineage was severely suppressed, demonstrating mild megaloblastoid changes. Megakaryocytes were significantly reduced, with occasional micromegakaryocytes present. A peripheral blood smear identified 1 blast per 50 leukocytes.

#### 2.4.2 Flow cytometry immunophenotyping

Flow cytometry demonstrated an expansion of monocytes, including approximately 2% immature monocyte-like cells displaying aberrant immunophenotypes. Granulocytes showed abnormal antigen expression patterns, and 2.5% aberrant myeloid blasts were quantified.

#### 2.4.3 BM biopsy and immunohistochemistry

Core biopsy findings aligned with a myelodysplastic neoplasm with low blasts (MDS-LB). Reticulin fibrosis was graded as MF-0, and iron staining was positive. IHC staining showed: CD34 (~2% blasts), CD117 (~2% blasts), MPO (positive in granulocytic lineage), CD61 (positive in megakaryocytes), CD71 (positive in erythroid lineage), and a Ki-67 index of ~60%. Scattered T cells (CD3^+^/CD5^+^), rare B cells (CD20^+^/PAX5^+^), and scattered plasma cells (CD138^+^) were noted.

#### 2.4.4 Cytogenetic and FISH analysis

Fluorescence *in situ* hybridization (FISH) using an MDS panel was positive for the *TP53*/CEP17 deletion, while D8Z2, D5S23/D5S721/CSF1R, D7Z1/D7S486, and D20S108 were negative. Conventional cytogenetic analysis revealed a highly complex karyotype strictly adhering to the International System for Human Cytogenomic Nomenclature (ISCN): 46, XX[13]/46, X, -X, add(5)(p15), +mar[3]/45, XX, -17, der(19)t(17;19)(q11.2;p13.1), add(21)(q22)[2]/45, XX, der(17;21)t(17;21)(p13;q22)?, ins(21);?(q22);?[2].

Correlating her extensive history of cytotoxic exposure with these morphological, molecular, and cytogenetic findings, a formal diagnosis of therapy-related myelodysplastic syndrome (t-MDS) was established.

### 2.5 Rapid leukemic transformation (april 2026)

In April 2026, approximately two months after the initial t-MDS diagnosis, the patient returned with worsening fatigue and was rehospitalized. A repeat bone marrow evaluation was performed:

#### 2.5.1 BM morphology

The marrow cellularity had shifted to relatively active, with an M:E ratio of 6.1:1. The smear was dominated by approximately 35% abnormal cells. These blasts varied in size, displayed a moderate amount of blue-gray cytoplasm, and occasionally exhibited fine cytoplasmic granules. Nuclear chromatin was delicate and uniform, with distinct nucleoli. Myeloperoxidase (MPO) staining showed partial positivity. Granulocytic, erythroid, and megakaryocytic lineages remained severely suppressed with persistent dysplastic features. The peripheral blood smear revealed 2% circulating blasts.

#### 2.5.2 Flow cytometry/minimal residual disease panel

Analysis revealed that monocytes were markedly increased, constituting 40.44% of all nucleated cells. Among them, mature monocytes (CD64^+^CD14^+^) and immature monocytes (CD64^+^CD14^-^) accounted for 27.36% and 13.08% of all nucleated cells, respectively. Abnormal myeloid blasts accounted for 25.63% of nucleated cells, displaying an aberrant immunophenotype characterized by CD45^dim^, positive expression of CD34, CD117, CD13, CD33, CD38, HLA-DR, and CD64, as well as partial expression of CD15 and CD56, and rare expression of CD17.

These findings confirmed rapid transformation into therapy-related acute myeloid leukemia (t-AML) evolving from t-MDS.

### 2.6 Salvage management and clinical outcome

Following the diagnosis of t-AML, a low-intensity salvage regimen comprising azacitidine and venetoclax was initiated. However, within 48 hours of starting therapy, the patient’s cytopenias worsened critically, and she developed a high fever indicative of neutropenic sepsis. Following infectious disease consultation, broad-spectrum antimicrobial therapy was adjusted. Due to her poor performance status, profound bone marrow failure, and uncontrolled infection, venetoclax was discontinued, and chemotherapy was suspended. The patient was transitioned to optimized best supportive care and anti-infective management.

## 3 Discussion

Therapy-related myeloid neoplasms (t-MN) are highly lethal clonal malignancies that arise as long-term sequelae of cytotoxic chemotherapy and/or radiation therapy, accounting for roughly 10%–20% of all diagnosed cases of MDS and AML (8). The patient in this report possessed multiple established risk factors for t-MN development, including advanced age, extensive exposure to alkylating agents and topoisomerase II inhibitors across multiple lines of salvage chemo-immunotherapy, localized radiotherapy, and subsequent CAR-T cell therapy (6).

Notably, t-MN secondary to topoisomerase II inhibitors typically manifests with a short latency period of 2 to 3 years post-treatment and frequently harbors balanced chromosomal translocations targeting 11q23 or 21q22 (4). In our patient, structural abnormalities involving chromosome 21q22 were identified, while additional lesions affecting chromosomes 5 and 17, together with a TP53 mutation, contributed to a highly complex karyotype. Chromosome 5 abnormalities are commonly observed in alkylating agent–related myeloid neoplasms, whereas alterations involving 21q22 may reflect genomic regions frequently targeted in topoisomerase II inhibitor–associated leukemogenesis. Taken together, these findings suggest that the patient’s disease likely resulted from cumulative genomic injury related to multiple prior therapeutic exposures rather than a single leukemogenic mechanism.

With the rapid global adoption of CAR-T cell therapy for hematologic malignancies, differentiating prolonged post-CAR-T hematotoxicity from an emerging secondary malignancy has become an urgent clinical dilemma. Severe and protracted cytopenia following CAR-T infusion is highly prevalent and usually attributed to several overlapping non-malignant mechanisms: (i) exhaustion of the hematopoietic stem cell reserve caused by repetitive prior lines of heavy chemotherapy; (ii) direct bone marrow microenvironmental injury secondary to lymphodepleting conditioning regimens; and (iii) prolonged, cytokine-mediated suppression of hematopoiesis stemming from immune effector cell-associated neurotoxicity syndrome (ICANS) or cytokine release syndrome (CRS) (9). To risk-stratify patients predisposed to severe hematotoxicity, the CAR-HEMATOTOX score was recently developed (10). The CAR-HEMATOTOX score, retrospectively calculated on October 11, 2025 during follow-up for persistent cytopenia after CAR-T cell infusion on May 8, 2025, was high (>2), driven by a markedly elevated baseline ferritin level (1367.31 ng/mL) and profound thrombocytopenia (58 × 10^9^/L).

Emerging genomic evidence suggests that CAR-T therapy and its associated lymphodepleting regimens may act as potent selective pressures that promote the expansion of pre-existing clonal hematopoiesis of indeterminate potential (CHIP), particularly clones harboring TP53 mutations. Rather than directly inducing *de novo* leukemogenic mutations, CAR-T–associated inflammatory stress and lymphodepletion may preferentially deplete normal hematopoietic stem and progenitor cells, thereby creating a permissive microenvironment that favors the competitive expansion of pre-existing mutant clones. In the present case, the identification of a TP53 mutation together with a complex karyotype at the time of t-MDS diagnosis is consistent with this proposed mechanism. However, pre-treatment molecular analyses were not available; therefore, the presence of a TP53-mutated clone prior to CAR-T therapy could not be directly confirmed. Consequently, this hypothesis should be interpreted with caution and requires validation in future longitudinal studies incorporating serial genomic monitoring.

A defining characteristic of this case was its remarkably aggressive biology: the disease transitioned from therapy-related myelodysplastic syndrome with low blasts (t-MDS-LB) (13% bone marrow blasts) to overt t-AML (35% blasts) within a two-month window. Although diagnostic criteria distinguish MDS and AML based on a 20% blast threshold, TP53-mutated t-MN frequently exhibits rapid biological instability, rendering these entities a continuum rather than discrete stages. Accordingly, the observed progression likely reflects intrinsic clonal evolution driven by TP53 dysfunction and complex cytogenetics rather than *de novo* leukemogenesis. This rapid transformation highlights the clinical futility of relying solely on routine peripheral blood monitoring when cytopenias persist abnormally. Clinicians must maintain a low threshold for initiating early, serial bone marrow evaluations—including cytogenetics and molecular testing—especially when faced with transfusion-refractory thrombocytopenia or atypical dysplastic signs on a peripheral smear. The discrepancy between morphologic and flow cytometric blast percentages (13% vs 2.5%) likely reflects hemodilution of the bone marrow aspirate submitted for immunophenotypic analysis, a known pre-analytical limitation that can result in underestimation of blast burden in flow cytometry compared with morphologic assessment of particle-rich smears.

Therapeutic avenues for elderly patients diagnosed with *TP53*-mutated t-MDS/AML remain incredibly restricted (11). Although the combination of hypomethylating agents (HMAs) and venetoclax is the established standard of care for older adults unfit for intensive chemotherapy, response rates remain dismal and transient in patients with complex cytogenetics and *TP53* deficiencies. As observed in this case, the profound baseline marrow failure characteristic of t-MN severely limits tolerability, frequently resulting in fatal infectious complications or prolonged cytopenias that necessitate premature treatment discontinuation. Consequently, there is an urgent need for novel therapeutic strategies, such as *TP53*-targeting small molecules or innovative immunotherapies, to improve outcomes in this ultra-high-risk patient population.

This case has several strengths, including comprehensive longitudinal clinical follow-up and integrated morphological, flow cytometric, cytogenetic, and molecular characterization across disease stages. Nevertheless, important limitations should be acknowledged. First, the absence of pre–CAR-T molecular data limits definitive assessment of pre-existing clonal hematopoiesis. Second, as a single-case observation, causal inference between CAR-T–related factors and clonal evolution cannot be established. Third, cumulative prior cytotoxic exposure likely represents a major confounding contributor to leukemogenesis.

In summary, this case underscores that persistent cytopenia after CAR-T therapy should not be attributed solely to treatment-related hematotoxicity (12). Early and repeated bone marrow evaluation, incorporating cytogenetic and molecular studies, is essential in high-risk patients to avoid delayed recognition of secondary myeloid malignancies (13). This report further highlights the importance of integrating dynamic clinical, morphological, and genomic data to distinguish hematotoxicity from evolving therapy-related clonal disease in the era of CAR-T therapy.

## 4 Conclusion

In conclusion, this case illustrates the diagnostic challenge of distinguishing post–CAR-T hematotoxicity from emerging therapy-related myeloid neoplasms in heavily pretreated patients. While predictive indexes like the CAR-HEMATOTOX score are invaluable for managing expected toxicities, they should not desensitize clinicians to the possibility of secondary malignancies. Prolonged, unexplained cytopenias in patients with intensive exposure to topoisomerase II inhibitors and alkylating agents warrant early, proactive diagnostic marrow evaluations. Distinguishing these entities promptly is paramount to adjusting therapeutic expectations and exploring novel investigational pipelines for t-MN.

## Data Availability

The original contributions presented in the study are included in the article/supplementary material. Further inquiries can be directed to the corresponding author.
